# Cerebral venous thrombosis in a patient with Down syndrome and coronavirus disease 2019: a case report

**DOI:** 10.1186/s13256-021-02908-0

**Published:** 2021-07-12

**Authors:** Henry Robayo-Amortegui, Natalia Valenzuela-Faccini, Cesar Quecano-Rosas, Darlyng Zabala-Muñoz, Michel Perez-Garzon

**Affiliations:** 1 Critical Medicine and Intensive Care Resident, Universidad de la SabanaFundación Clínica Shaio, Bogotá, Colombia; 2 Intensive Care Specialist, Fundación Clínica Shaio, Bogotá, Colombia

**Keywords:** Down syndrome, COVID-19, Cerebral venous thrombosis, Coagulopathy, Neuroinfection diseases, Neurological complications

## Abstract

**Background:**

The new coronavirus disease 2019 pandemic has spread throughout most of the world. Cerebral venous thrombosis is a rare thromboembolic disease that can present as an extrapulmonary complication in coronavirus disease 2019 infection.

**Case presentation:**

We report the case of a Hispanic woman with Down syndrome who has coronavirus disease 2019 and presents as a complication extensive cerebral venous thrombosis.

**Conclusions:**

Cerebral venous thrombosis is a rare thromboembolic disease that can present as an extrapulmonary complication in coronavirus disease 2019 infection. In the absence of clinical and epidemiological data, it is important to carry out further investigation of the risk factors and pathophysiological causes related to the development of cerebrovascular thrombotic events in patients with Down syndrome with coronavirus disease 2019 infection**.**

## Introduction

The new coronavirus disease 2019 (COVID-19) pandemic has spread throughout most of the world, causing more than 28 million infections to date, with more than 1 million deaths worldwide [1]. This infection is mainly associated with lung compromise, which causes pneumonia and acute respiratory distress syndrome; however, recent studies have described associations with prothrombotic states favored by a hypercoagulable state compromising the perfusion of vital organs such as the lung and central nervous system. Regarding the latter, the incidence of cerebrovascular events in hospitalized patients with COVID-19 is reported to be at 0.5%, with venous sinus thrombosis being the most infrequent clinical presentation in this group [2]. We report the case of a woman with Down syndrome who presents with COVID-19 and presents as a complication extensive sinus venous thrombosis.

## Case report

We report the case of a 36-year-old Hispanic woman whose medical history features Down Syndrome, grade 1 obesity, and hypothyroidism in hormonal supplementation without a history of epilepsy or seizure. She was admitted to the emergency department with 7 days of diarrhea without mucus or blood, dry cough, progressive altered mental status, and an episode of focal tonic–clonic seizure in the left half of the body, which later became generalized. Upon arrival, the patient’s vital signs showed a blood pressure of 110/60 mmHg, heart rate of 115 beats/minute, respiratory rate 30 breaths/minute, and oxygen saturation of 88% with supplementary oxygenation with a high mask flow of 15 L. On physical examination, her Glasgow Coma Scale score was 11/15, and she showed drowsiness and poor interaction with the examiner. She presented reactive isochoria, pulmonary auscultation with rales in both lung fields with signs of respiratory distress given by polypnea, and use of accessory muscles. During the evaluation, she had a grand mal seizure that progressed to status epilepticus, requiring management with diazepam and valproic acid, airway protection, and invasive ventilatory support. Laboratory findings reported leukocytosis with neutrophilia and lymphopenia and elevated lactate dehydrogenase and D-dimer (18,320 ng/mL). Arterial gases showed respiratory alkalosis with severe hypoxemia and hyperlactatemia, severe acute respiratory syndrome coronavirus 2 (SARS-CoV-2) was confirmed with reverse transcription polymerase chain reaction (RT-PCR), and chest X-ray showed peripheral interstitial opacities in both lung fields.

**Fig. 1:**
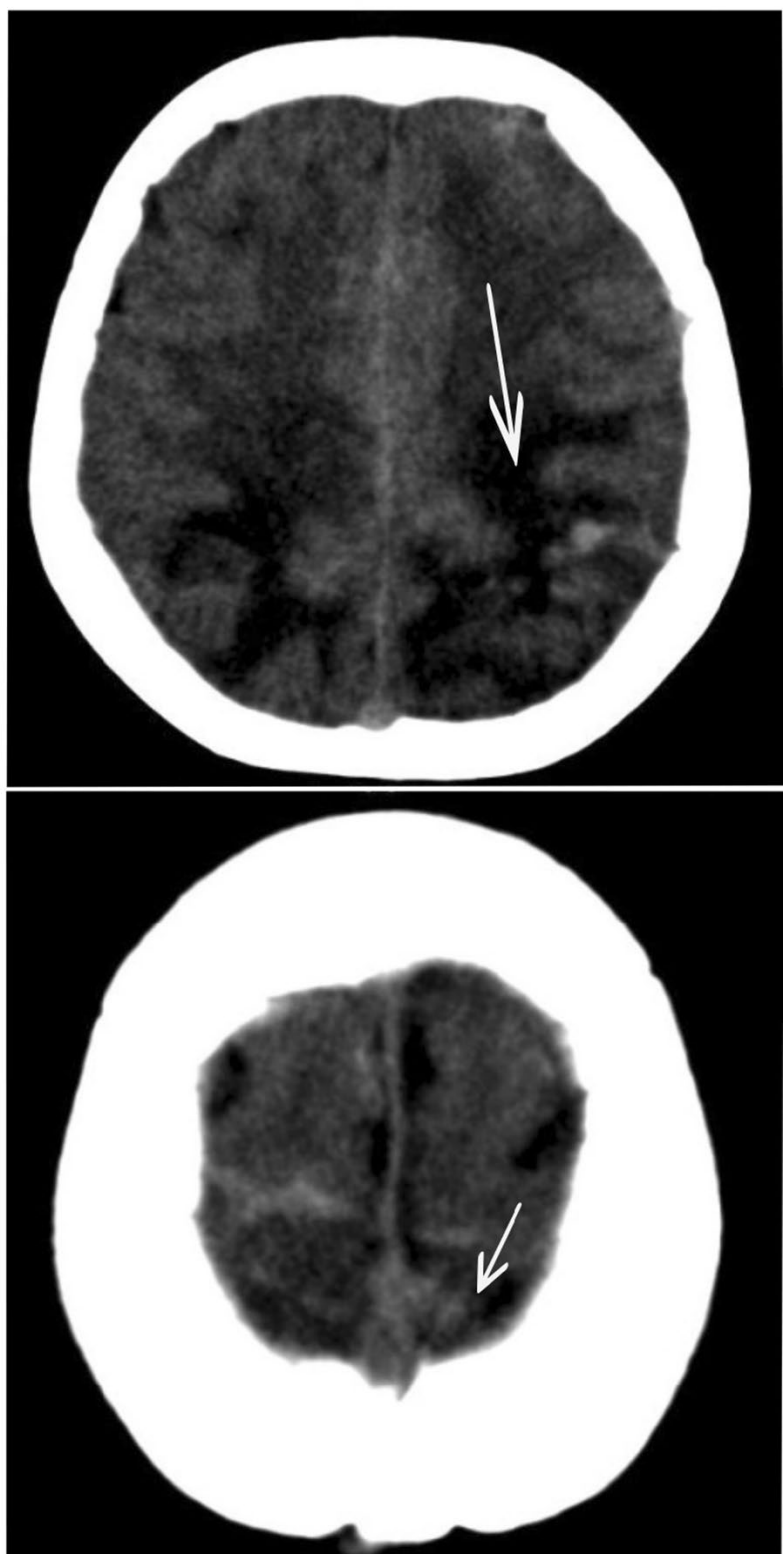
Simple tomography: The arrow points at vasogenic edema in parietooccipital regions of both cerebral hemispheres, with extension to the posterior region of the frontal lobes, small foci of subcortical hemorrhage on the left side. Asymmetric collapse of the ventricular atria with anterior deviation of the left

**Fig. 2:**
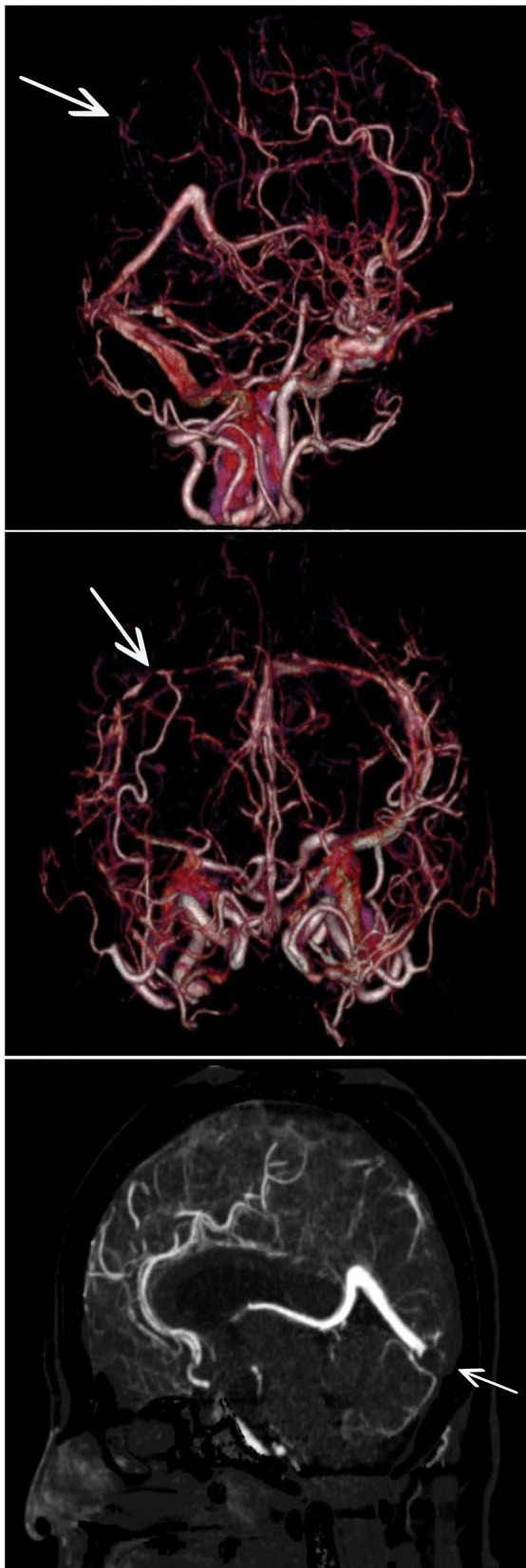
Cerebral CT angiography: The arrow points at extensive venous thrombosis withocclusion of the entire superior sagittal sinus, torcula, and terminalportion of the rectus sinus. Subtotal occlusion of transverse sinuses

She was transferred to the intensive care unit, where treatment for refractory hypoxemia was initiated with protective mechanical ventilation and prone therapy. A simple brain computed tomography (CT) scan was performed, reporting left parietal intraparenchymal hemorrhage with associated vasogenic edema, without any evidence of drainage in the ventricles or signs of intracranial hypertension; thus, neuroprotection was initiated, and management with valproic acid and levetiracetam was continued. Due to the findings described, the initiation of anticoagulation was postponed. On the second day in the intensive care unit (ICU), due to persistent bradycardia and progressive elevation of D-dimer to 25,000 ng/mL, a simple CT brain scan was performed reporting indirect signs of thrombosis of the superior sagittal sinus with venous infarcts (Fig. 1). Afterwards, cerebral CT angiography with venous phase confirmed extensive venous thrombosis of superior sagittal sinus, herophilic torcula, and terminal portion of the rectus sinus, as well as subtotal occlusion of transverse sinuses (Fig. 2). Hence, full anticoagulation was started with low-molecular-weight heparin (LMWH) at 1 mg/kg every 12 hours. Further analyses on prothrombotic disease were performed such as tests for lupus anticoagulant, anticardiolipin antibodies immunoglobulin G (IgG) and immunoglobulin M (IgM), beta 2 glycoprotein IgM and IgG, and chorionic gonadotropin, which were negative and ruled out pregnancy or antiphospholipid syndrome considering that the thrombotic event was secondary to COVID-19 infection.

**Fig. 3:**
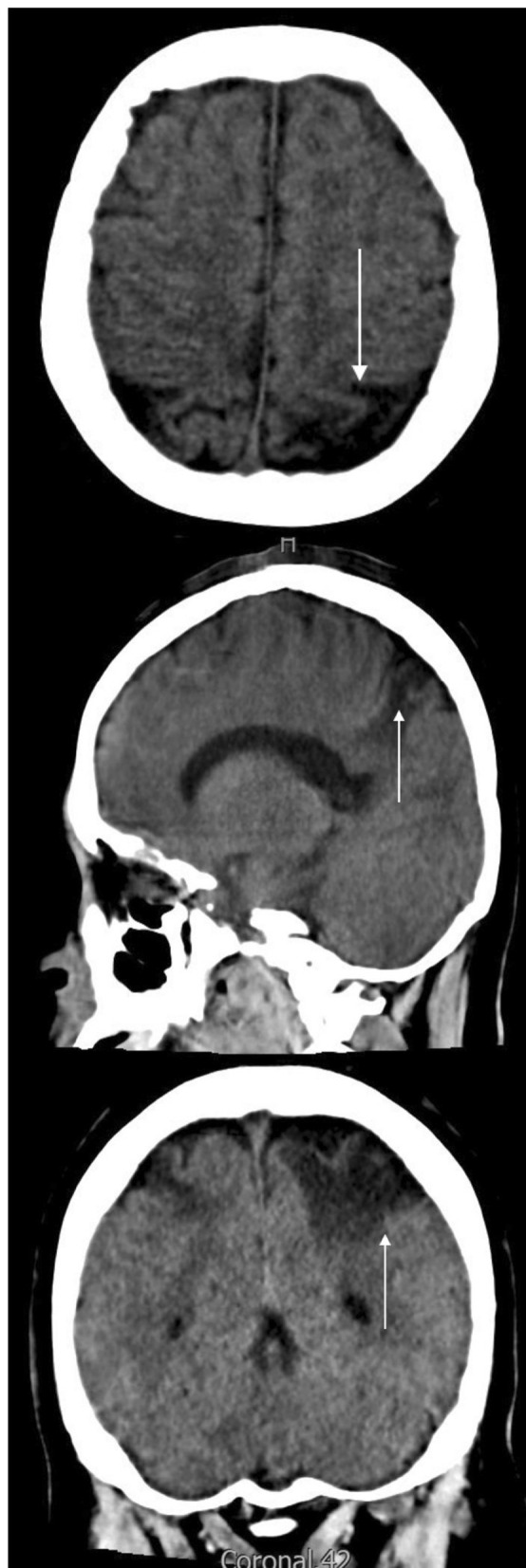
Six-month control brain CT scan: The arrow points at bilateral cortico-subcorticalareas of encephalomalacia in parietal lobes, with more extent in theleft lobe and small bands of cortical encephalomalacia in occipital convexities

During her stay in the ICU, she was under mechanical ventilation for 16 days and completed two cycles of prone therapy with improvement in oxygenation without signs of neurological deficit, so the sedation was suspended and she was successfully extubated. Afterward, she was transferred to a general ward for comprehensive rehabilitation, where no neurological sequelae were identified. At discharge, the anticoagulation was switched from LMWH to warfarin at a dosage of 5 mg per day 6 months after her the initial event a brain CT scan was performed as well as a neurological assessment that reported no neurological deficit. Since discharge, no seizures have been reported (Fig. 3).

## Discussion

The incidence of thromboembolic complications due to COVID-19 in ICU patients is close to 31% [3]. Among those, cerebral venous thrombosis is a rare presentation of a cerebrovascular event, with an approximate incidence of three to four cases per million people. The most frequent presentation is described for fertile women with risk factors for a prothrombotic condition such as hereditary, acquired, autoimmune, or infectious [4]. To date, no cases of CVT with compromise of the sigmoid sinuses and transverse sinuses have been reported in patients with Down syndrome associated with COVID-19 infection.

The hypercoagulability state caused by COVID-19 has generated cerebrovascular events as a complication in infected patients. In a meta-analysis carried out by Shahjouei *et al.* evaluating 17,799 hospitalized patients with COVID-19, the risk of a cerebrovascular event was described in 0.9% of cases, the most common being the ischemic event (79%), followed by intracranial hemorrhage (17%) and the most infrequent, sinus venous thrombosis (4%). The presentation of sinus venous thrombosis in COVID-19 is described to be more common in woman with a median age of 54 (± 39–58) years, presenting neurological symptoms at an average of 4 days from the onset of respiratory symptoms, which was evidenced in this case report in relation to the time of presentation of respiratory symptoms and the development of neurological symptoms (5 days) [2]. Nonetheless, in our case, it is worth highlighting the association of COVID-19 infection with a cerebral thrombotic event in a young adult woman with obesity and Down syndrome.

As previously described, COVID-19 infection presents multisystemic manifestations mainly pulmonary-associated with endotheliopathy as a pathophysiological basis. These manifestations can be explained through inflammatory and thrombotic pathways by binding to angiotensin-converting enzyme 2 receptors of endothelial cells, leading to endotheliitis and a diffuse procoagulant state [5]. Nevertheless, in patients with Down syndrome, several risk factors for developing severe infections have been described, such as dysregulation of the immune system due to the overexpression of genes encoded on chromosome 21 with immune regulation functions by the interferon receptors IFNAR1 and IFNAR2, and IFNGR2 and IL10RB that serve as ligands for cytokines interleukin (IL)-10, IL-22, and IL-26. Overexpression of these genes indicates that the response of interferon to viral infection leads to the amplification of cytokine storm in patients with Down syndrome, and thus, this phenomenon may be related to an increased prothrombotic risk [6].

The neurological manifestations of cerebral venous thrombosis include headache (87%) followed by seizures (34%), nausea or vomiting (33%), and an altered state of consciousness (21%) in fertile women. In our case, the patient presented with seizures and altered mental status that, according to the VENOST study in the subgroup of women of fertile age, corresponds to the second and fourth most frequent neurological manifestation, respectively [7]. In patients with Down syndrome, there is a higher prevalence of epilepsy than in the general population and between 1% and 23% present symptoms at 30 years, with myoclonic seizures being the main cause of late epilepsy in 40% of the patients, not focal or status epilepticus as in our patients. For the above, during the examination and when obtaining medical history, it is important to question neurological symptoms and seizures [8]. To date, there are no studies relating Down syndrome and cerebral venous thrombosis.

Regarding the diagnostic imaging performed, magnetic resonance imaging (MRI) was not considered since the diagnosis could be made with simple chest scan and angiotomography; simple axial tomography can show specific signs of cerebral venous thrombosis in up to 30% of cases, such as the dense triangle sign and the cord sign. If the diagnosis is suspected, it should be confirmed with computed tomography plus venography, which can show absence of partial or complete flow in thrombosed veins or sinuses [9]. The guidelines of the European Stroke Organization suggest magnetic resonance venography or computed tomography plus venography to confirm the diagnosis [10]. A meta-analysis by Xu *et al.* compared 48 studies to systematically evaluate the performance of chest scan and MRI in the diagnosis of cerebral venous thrombosis, finding that computed tomography plus venography [OR 57.18 IC (35.59, 91.87)] and magnetic resonance venography [OR 38.34 (13.77, 106.77)] have a high level of diagnostic precision in cerebral venous thrombosis [11].

In terms of the management of cerebral venous thrombosis, anticoagulation is the treatment of choice as soon as the diagnosis is made. When using low-molecular-weight heparin and unfractionated heparin, no significant difference was found with respect to bleeding, mortality, and morbidity outcomes [12]. According to the rating scale for cerebral venous thrombosis from Barbosa *et al.*, our patient had a score of 6 for a 30-day mortality risk of 10%; however, the outcome presented in the patient at 3-month follow-up from the onset of symptoms showed no complications or sequelae related to cerebral venous thrombosis, or fatal outcomes [13].

## Conclusion

In conclusion, cerebral venous thrombosis is an infrequent thromboembolic disease, presented as an extrapulmonary complication in COVID-19 infection, which manifests itself mainly in fertile women, with headache being the most common symptom. Diagnosis can be made by computed tomography plus venography or magnetic resonance venography. Currently, there are no epidemiological data or pathophysiological causes related to the development of cerebral venous thrombosis in patients with Down syndrome with COVID-19 infection.

## Data Availability

Not applicable.

## Abbreviations

COVID-19: Coronavirus disease 2019
ARDS: Acute respiratory distress syndrome
DS: Down syndrome
LDH: Lactate dehydrogenase
ICU: Intensive care unit
CT: Computed tomography
LMWH: Low-molecular-weight heparin
CVT: Cerebral venous thrombosis
ACE II: Angiotensin-converting enzyme 2 receptors
ESO: European Stroke Organization
MRV: Magnetic resonance venography
UFH: Unfractionated heparin

## References

[1] World Health Organization. Coronavirus disease 2019 (COVID-19): Dashboard 13th May, 2020. https://covid19.who.int/. Accessed 1 Oct 2020.

[2] Shahjouei S, Naderi S, Li J, *et al*. Risk of stroke in hospitalized SARS-CoV-2 infected patients: a multinational study. EBioMedicine. 2020;59:102939. 10.1016/j.ebiom.2020.102939 PMC7429203 32818804

[3] Klok FA, Kruip MJHA, van der Meer NJM, *et al*. Incidence of thrombotic complications in critically ill ICU patients with COVID-19. Thromb Res. 2020;191:145–7. 10.1016/j.thromres.2020.04.013 PMC7146714 32291094

[4] Idiculla PS, Gurala D, Palanisamy M, *et al*. Cerebral venous thrombosis: a comprehensive review. Eur Neurol 2020; 83:369–79. 10.1159/000509802 32877892

[5] Libby P, Lüscher T. COVID-19 is, in the end, an endothelial disease. Eur Heart J. 2020;41:3038–44. 10.1093/eurheartj/ehaa623 PMC7470753 32882706

[6] Espinosa JM. Down syndrome and COVID-19: a perfect storm? Cell Rep Med. 2020;1:100019. 10.1016/j.xcrm.2020.100019 PMC7252041 32501455

[7] Uluduz D, Sahin S, Duman T, *et al*. Cerebral venous sinus thrombosis in women: subgroup analysis of the VENOST Study. Stroke Res Treat. 2020; 8610903. 10.1155/2020/8610903 PMC7481993 32953038

[8] Barboza MA, Chiquete E, Arauz A, *et al*. A practical score for prediction of outcome after cerebral venous thrombosis. Front Neurol. 2018;9:882. 10.3389/fneur.2018.00882 PMC6204492 30405516

[9] Ulivi L, Squitieri M, Cohen H, Cowley P, Werring DJ. Cerebral venous thrombosis: a practical guide. Pract Neurol. 2020;20(5):356–67. 10.1136/practneurol-2019-002415 32958591

[10] Coutinho JM, Crassard I, Dentali F, *et al*. European Stroke Organization guideline for the diagnosis and treatment of cerebral venous thrombosis – endorsed by the European Academy of Neurology. Eur J Neurol 2017;24:1203–13. 10.1111/ene.13381 28833980

[11] Xu W, Gao L, Li T, *et al*. The performance of CT versus MRI in the differential diagnosis of cerebral venous thrombosis. Thromb Haemost 2018;118:1067–77. 10.1055/s-0038-1642636 29695023

[12] Qureshi A, Perera A. Low molecular weight heparin versus unfractionated heparin in the management of cerebral venous thrombosis: a systematic review and meta-analysis. Ann Med Surg (Lond). 2017;17:22–6. 10.1016/j.amsu.2017.03.016 PMC5376268 28392913

[13] Santoro JD, Pagarkar D, Chu DT, *et al*. Neurologic complications of down syndrome: a systematic review. J Neurol. 2020. 10.1007/s00415-020-10179-w 32920658

